# To Our Readers

**Published:** 1875-08

**Authors:** 


*To our Readers:*

Overhaul your case-books and send us your experience. We desire original articles, reports of societies, clinics, news, etc. Our columns are open to all. We ask all of our readers to aid us in making the RECORD the best journal for practical purposes ever published in this country.

